# Control of nucleolar stress and translational reprogramming by lncRNAs

**DOI:** 10.15698/cst2019.01.172

**Published:** 2018-12-05

**Authors:** Yvessa Verheyden, Lucas Goedert, Eleonora Leucci

**Affiliations:** 1 Laboratory of RNA Cancer Biology, Department of Oncology, LKI, KU Leuven, 3000 Leuven, Belgium.; 2 Department of Cell and Molecular Biology, Faculty of Medicine of Ribeirão Preto, University of São Paulo, Ribeirão Preto, São Paulo, Brazil.

**Keywords:** lncRNAs, ISR, IRES, uORFs, nucleolus, stress response, adaptation, ribosome biogenesis

## Abstract

Under adverse environmental conditions, cells activate stress re-sponses that favour adaptation or, in case of irreversible damage, induce cell death. Multiple stress response pathways converge to downregulate ribo-some biogenesis and translation since these are the most energy consuming processes in the cell. This adaptive response allows preserving genomic stabil-ity and saving energy for the recovery. It follows that the nucleolus is a major sensor and integrator of stress responses that are then transmitted to the translation machinery through an intricate series of conserved events. Long non-coding RNAs (lncRNAs) are emerging as important regulators of stress-induced cascades, for their ability to mediate post-transcriptional responses. Consistently, many of them are specifically expressed under stress conditions and a few have been already functionally linked to these processes, thus fur-ther supporting a role in stress management. In this review we survey differ-ent archetypes of lncRNAs specifically implicated in the regulation of nucleo-lar functions and translation reprogramming during stress responses.

## INTRODUCTION

Unfavourable environmental conditions trigger cellular responses that help minimize and/or repair the damage experienced by the cell. During stress responses, most of the biosynthetic processes of the cell are reduced or shut down, to spare energy and preserve genome integrity, while selected pathways that help the recovery are specifically activated. Occurring in a contest of global transcriptional repression, responses to stress often involve epigenetic and posttranscriptional events [1].

Ribosomes, the core components of the translation apparatus, are molecular machineries composed by four different ribosomal RNAs (rRNAs) and about 79 ribosomal proteins. Ribosome biogenesis is initiated in the nucleolus with the transcription of the rRNAs, and requires the coordinated activity of all the three polymerases together with a large number of transcription factors, nucleases and small RNAs that contribute to the processing and maturation of the rRNAs [2]. Utilizing about 80% of the cellular total energy, ribosome biogenesis is tightly regulated by the availability of nutrients and directly connected to cell cycle regulation, cell growth and apoptosis [3]. It follows that the nucleolus is a major sensor of stress and that the exposure to different stressors, including DNA damage, heat shock, hypoxia, viral infection, and others, all converge to downregulate and rewire ribosome biogenesis and translation [4]. Integration of environmental signals with ribosome biogenesis and translation is mostly achieved through the mTOR pathway (**BOX1**). Favorable extracellular conditions are translated by mTOR into cellular mass through phosphorylation of two main targets: 4EBP1 and S6K. While phosphorylation of this last one induces transcription of the rRNAs and boosts ribosome biogenesis, phosphorylation of 4EBP1 results into the release of eIF4E that become available for binding with other initiation factors, thus leading to translation initiation [5]. Different stressors, not only affect the synthesis of functional ribosomes, but also regulate the rates of protein synthesis by triggering the so-called integrated stress response or ISR [6]. The ISR is triggered by different kinase receptors each activated by a different stimulus (e.g., viral infection, amino acid deprivation, ER stress…) and all converging to phosphorylation of eIF2alpha, that blocks CAP-dependent translation in favour of ATF4 activation and non-canonical translation [6]. Translational rewiring upon stress is a key driver of cell plasticity and as such it is essential for many biological processes including regulation of lifespan [3] and cancer progression [7–9], therefore a deep understanding of this process will have important biological and therapeutic implications.

BOX 1 **The mTOR pathway.** The mammalian Target of Rapamycin (or mTOR) is a member of the phosphatidylinositol 3-kinase (PI3K)-related kinases. mTOR integrates the intracellular energetic status (ATP/AMP ratio) and the availability of nutrients and growth factors to activate cell metabolism and promote cell growth. Specifically, mTOR promotes *de novo* synthesis of lipids, nucleotides and proteins while inhibiting autophagy. mTOR exists in two different complexes the TOR complex 1 (TORC1) and the TORC2 complex. Differently from complex 1 the TORC2 complex can be activated only by growth factors and the details of its regulation is poorly understood. Both complexes however are downstream of the PI3K pathway [52].

TORC1 promotes **global translation** by phosphorylating the translational inhibitor 4E-BP, an event that causes its release from the initiation factor 4E (eIF4E). Furthermore, TORC1 is responsible for the translation of a subset of RNAs containing a 5' OligoPyrimidine Tract (5'TOP) encoding for translational machinery components. Lastly, by phosphorylation of anoth-er target, the S6K, TORC1 controls **ribosome biogenesis**. Following TORC activation, S6K phosphorylates the ribosomal protein S6 and CAD (Gln-dependent carbamoyl-phosphate synthase, Asp carbamoyltransferase, dihydroorotase). This last event stimulates pyrimidine synthesis and thus rRNA biosynthesis [53].

Modulation of **autophagy** is mostly achieved by phosphorylation and inactivation of the pro-autophagic UNC-51-like ki-nase 1 (ULK1) [54].

**Lipogenesis** is under control of both mTORC1 and mTORC2, through phosphorylation of SREBPs, the master regulators of lipogenic genes. Specifically, SREBPs phosphorylation is mediated by S6K and AKT respectively [55].

The last decade has witnessed an increased interest in lncRNAs, transcripts longer that 200 nucleotides that do not encode for proteins. Due to their pivotal role in the regulation of epigenetic and post-transcriptional events, they have been increasingly linked to stress responses. Here we summarize and discuss the existing knowledge about the role of these fascinating molecules in nucleolar stress and translational rewiring (**Table 1**).

**TABLE 1.** LncRNAs archetypes regulating nucleolar functions and translation reprogramming during stress responses.

**Name**	**Function**	**Inducing stress**	**Reference**
Ribosome InterGenic Spacer (IGS)	Protein immobilization in nucleolus	Heat shock and acidosis	[13, 14]
Promoter And Pre-rRNA AntiSense (PAPAS)	rDNA silencing	Quiescence and hypotonic stress	[15, 16, 17]
SLERT	Pre-rRNA transcription promotion	Oncogenic stress	[18]
SAMMSON	rRNA maturation	Oncogenic stress	[19]
Circular ANRIL (circANRIL)	rRNA maturation	/	[21]
ZFAS1	Ribosome assembly regulation	Oncogenic stress	[39]
Uchl1	Translation promotion	/	[40]
BACE1-AS (BACE1)	mRNA and protein level regulation	Hyperthermia, serum starvation, Aβ 1–42, hydrogen peroxide or glucose shock	[41]
Natural Antisense RNA Zeb2 (Zeb2-NAT)	Alternative splicing regulation	Not known	[45]
Translational Regulatory lncRNA (treRNA)	E-Cadherin translation supression	Oncogenic stress	[46]
Neighbour of BRCA1 gene 2 (NRB2)	AMPK activity promotion	Energy stress	[47]

## LncRNAs in RIBOSOME BIOGENESIS

**Figure 1:**
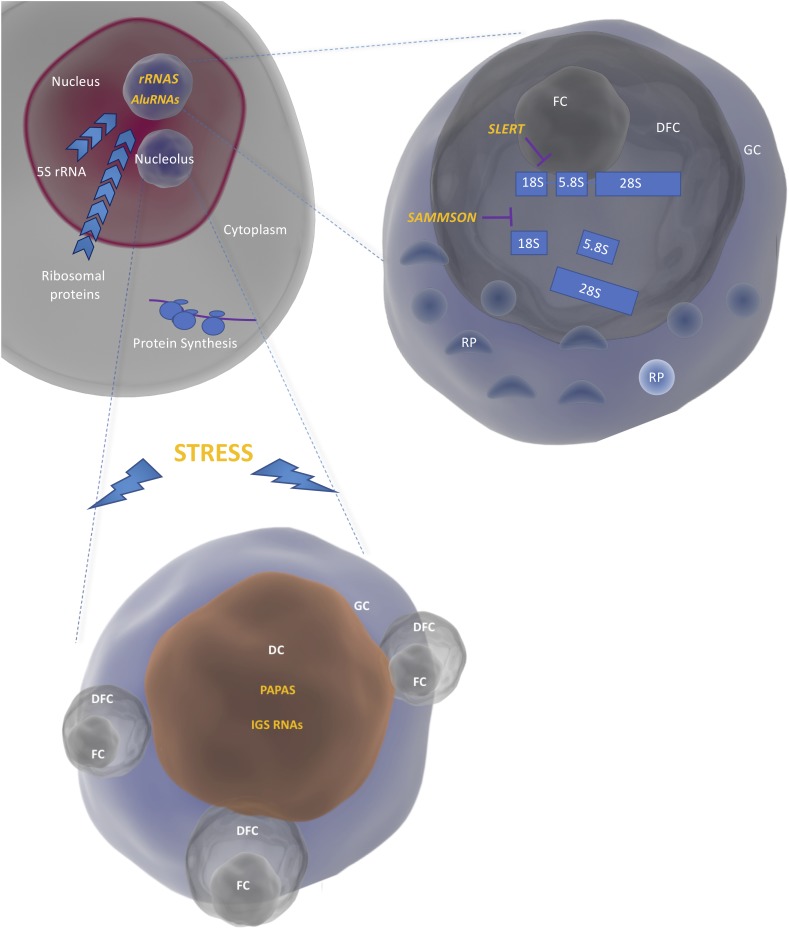
FIGURE 1: Compartmentalization of ribosome biogenesis in the nucleoli. The rRNAs are transcribed by the RNA polymerase I as a single precursor (47S) at the interface between the FC and the DFC. The mature rRNAs are produced in the DFC and assembled into large and small ribosomal subunits by binding to the ribosomal proteins (RP) coming from the cytoplasm and the 5S (produced in the nucleoplasm) in the GC. Interaction of rRNA and AluRNAs with proteins leads to the formation of nucleoli through liquid-liquid phase separation. Under conditions of stress, IGS RNAs and PAPAs are upregulated thus leading to nucleolar remodeling and rRNA transcriptional silencing respectively. Nucleolar remodeling results in the formation of DC (Detention Centers). *SLERT* and *SAMMSON* regulate rRNA transcription and processing respectively.

Nucleoli are membrane-less bodies within the nucleus in which the early steps of ribosome biogenesis take place. Nucleoli are divided into different compartments: the Fibrillar Center (FC), the Dense Fibrillar Components (DFC) and the Granular Component (GC). The transcription of rRNA genes by the RNA polymerase I takes place at the interface between FC and DFC, the resulting 47S precursor is then processed in the DFC and the ribosomal subunits are finally assembled in the in the GC (**Figure 1**). The structure and composition of the nucleoli are dynamically regulated during the cell cycle and profoundly altered by environmental stress [10].

LncRNAs can interfere with ribosome biogenesis in the nucleoli in three different ways: (i) by affecting nucleolar structure, (ii) by affecting rRNA and/or ribosomal protein production (iii) or by affecting rRNA and/or ribosomal protein maturation.

The nucleolar compartment assembles through phase separation of its molecular components and its sub-compartments are made of coexisting liquid phases [11]. Phase transition is caused by interaction of intrinsic disordered domain containing proteins, such as fibrillarin, nucleolin and nucleophosmin, with non-coding RNAs, thus underlining the importance of the RNA for the maintenance of nucleolar structure and function. Supporting this view, recombinant fibrillarin and nucleophosmin form liquid droplets when combined with rRNA *in vitro* and, if mixed, the droplets will recapitulate the structure of nucleoli [11]. In addition, Alu-related B1 RNAs, polymerase II transcripts of about 250 nucleotides, are also enriched in the nucleoli and essential for nucleolar integrity. A recent study revealed that overexpression of these transcripts is sufficient to increase the size of nucleoli and the pre-rRNA processing, while their depletion leads to dispersion of nucleoli [12].

Environmental stressors such as heat shock and acidosis cause lncRNA-dependent changes in nucleolar organization and structure. Jacob and colleagues showed that ribosome InterGenic Spacer (IGS) RNAs are induced upon stress, such as heat shock and acidosis, from very defined regions separating individual rDNA transcription units [13]. IGS RNAs target proteins containing NucleOlar Detention Sequences (NoDS) and immobilise them in the nucleolus [14]. Accumulation of IGS RNAs coincide with the formation of detention centers in the nucleoli and termination of rRNA transcription [13]. IGS RNAs are clear examples of ncRNAs affecting ribosome biogenesis through changes in nucleolar structure.

An example of lncRNAs affecting rRNA and/or ribosomal protein production, is the lncRNA Promoter And Pre-rRNA AntiSense (PAPAS), whose levels are increased during quiescence [15] and hypotonic stress [16]. During quiescence, PAPAS recruits Suv4-20h2, thus promoting trimethylation of histone H4 at lysine 20 (H4K20me3) and transcriptional silencing of rRNA genes [15]. Under the condition of hypotonic stress instead, when Suv4-20h2 is degraded, the same lncRNA causes nucleosome repositioning through the NuRD complex leading again to rDNA silencing [17].

A further example of a lncRNA regulating ribosome biogenesis is *SLERT*, a box H/ACA small nucleolar RNA (snoRNA)-ended lncRNA [18]. *SLERT* is expressed in embryonic stem cells and in a number of human cancer cell lines and requires H/ACA snoRNAs at both ends for its correct processing and localization to the nucleoli. *SLERT* promotes pre-rRNA transcription by binding to DEAD-box RNA helicase DDX21 and blocking its inhibitory activity on the RNA polymerase I. Inhibition of *SLERT* reduces tumorigenic potential *in vitro* and *in vivo* in xenograft models [18].

An example of the third category (lncRNA or by affecting rRNA and/or ribosomal protein maturation) comes from our laboratory. We recently showed that the lncRNA *SAMMSON* is required for proper rRNA maturation in the mitochondria and in the nucleus of melanoma cells. Its activity ensures the coordination of the two translation machineries to avoid the emergence of proteotoxic stress [19]. *SAMMSON* exerts its function by directly interacting with three major regulators of ribosome biogenesis in the two compartments: XRN2, CARF and p32. By favouring the formation of an aberrant complex between p32 and CARF, *SAMMSON* retains CARF in the cytosol and impairs the binding to its natural partner XRN2, in the nucleus. Since p32 and XRN2 are involved in ribosome biogenesis in the mitochondria and nucleoli respectively, by redirecting CARF affinities, *SAMMSON* affects their cellular localization and enhances concertedly their functions [19].

Recently also circular RNAs (circRNAs) [20], that derive from back splicing of coding and non-coding genes, have been implicated in the regulation of nucleolar functions. The lncRNA ANRIL, which is transcribed at chromosome 9p21, for example is able to form RNA circles (circANRIL). Expression of the circular form of *ANRIL* increases p53 expression and induces apoptosis [21]. Holdt *et al.* showed that circANRIL interacts with the protein pescadillo zebrafish homologue 1 (PES1), a component of the PES1-BOP1-WDR12 (PeBoWe) complex. qPCR analysis and Northern blotting showed an accumulation of 32S and 36S intermediates in circANRIL overexpressing cells. These findings all together suggest that circANRIL regulates rRNA maturation through PeBoWe complex and it could therefore trigger nuclear stress and p53 activation [21].

## ROLE OF lncRNAs IN TRANSLATIONAL REWIRING

**Figure 2:**
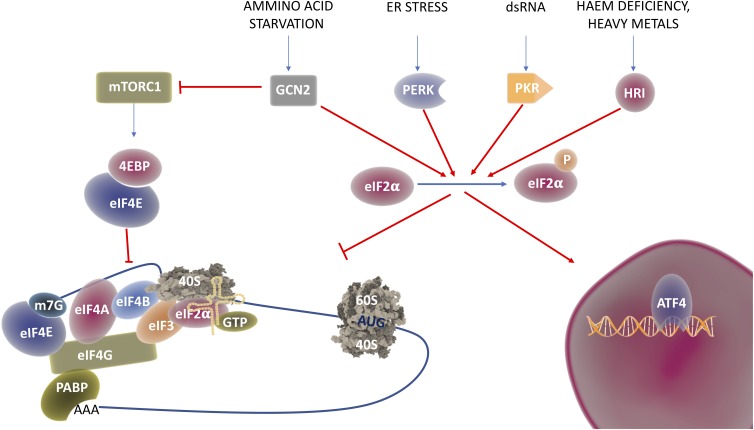
FIGURE 2: The ISR in translational reprograming. Environmental cues are able to turn off mTORC1, thus impeding phosphorylation of 4EBP and release of eIF4E, the initiation factor responsible for m7G cap recognition; this process leads to cap-dependent translational repression. In addition, different stimuli activate the stress sensor kinases GCN2, PERK, PKR or HRI that in turn phosphorylate eIF4α. Phospo ser51 eIF4α cannot exchange anymore GDP and GTP therefore formation of the ternary complex between the small ribosomal subunit, eIF4α and the initiator tRNAMeti is inhibited. While general translation is inhibited, special/selective translation is initiated by activation of the ATF4 transcriptional program.

Under conditions of stress, the cell undergoes translational reprograming, where global translation is downregulated while essential factors for the recovery are still synthetized through alternative translation pathways [22]. Shift from global to selective translation is mostly achieved by modification of the translation initiation process. Global translation, also known as cap-dependent translation, relies on the recognition of the 7-methylguanosine (m^7^Gppp) in the 5′-cap structure by eukaryotic initiation factor 4E and on the sequential assembly of initiation factors on the mRNA [23–25]. Under different stress conditions, stress sensing kinases (see **Figure 2**) get activated and prime the phosphorylation of Eukaryotic Initiation Factor 2 α (eIF2α) at serine 51, a condition that impairs the formation of the ternary complex necessary for cap-dependent translation [6]. In these conditions, different structures and sequences on the 5′ untranslated region (UTR) are used to recruit the ribosomes and ensure the translation of proteins required to overcome the stress. The most common way to activate cap-independent translation initiation are Upstream Open Reading Frames (uORFs) and Internal Ribosome Entry Site (IRES) [26, 27]. IRES are sequences in the 5′-UTR of coding transcripts, able to form complex structures that directly recruit the mRNAs to the ribosome without the need of most (and sometimes all) the initiation factors [24, 25]. Although first identified in viral RNAs [28], these sequences have been found also in the 5′-UTR of many oncogenes and tumor suppressors [29] including *c-Myc* [30], *p53* [31] and *Bcl2* [32].

Although lncRNAs are either not or only rarely translated into (small) peptides [33, 34], profiling of ribosome-associated RNAs has revealed that a large fraction of non-coding transcripts are associated with ribosomes [35–38]. This finding raises the intriguing possibility that several lncRNAs may contribute to the regulation of ribosome biogenesis and/or functions. LncRNA could affect translation in different ways: (i) by affecting ribosome assembly, (ii) by recruiting specific transcripts to the ribosome (ii) by masking (and unmasking) uORFs and/or IRES (iii) by interfering with translation factor activity or with specific signalling pathways.

Although these findings need further confirmation, *ZFAS1* could be an example of a lncRNA regulating ribosome assembly. *ZFAS1* was identified as a lncRNA binding to the light polysome fraction whose expression is induced during ribosome biogenesis in breast cancer cell [39].

Examples of lncRNAs regulating transcript recruitment to the ribosome are Uchl1 and *BACE1-AS*. It was recently demonstrated that the mouse lncRNA Uchl1 translocates to the cytoplasm upon mTORC1 activation, to promote translation of its own antisense transcript by base pairing to its 5′-end and enhancing recruitment of the ribosome [40]. *BACE1-AS*, the antisense to β-secretase 1 (*BACE1*), is elevated in brain of individuals with Alzheimer's disease [41]. *BACE1-AS* is able to stabilize the levels of *BACE1* mRNA and increase, through an unknown mechanism, the levels of β-secretase 1 in response to different stressors [41].

An elegant example of the third category is Natural Antisense RNA Zeb2 (*Zeb2-NAT*). An IRES sequence for instance is hidden in an intron in the 5′-UTR of *Zeb2*, a transcriptional repressor of E-cadherin and master regulator of epithelial-to-mesenchymal transition, an essential process during development [42] and cancer progression [43, 44]. Alternative splicing of the intron containing the IRES is regulated by *Zeb2-NAT* [45]. During the induction of EMT, *Zeb2NAT* gets upregulated and base-pairs to *Zeb2* mRNA, thus causing intron retention. Inclusion of the IRES sequence increases Zeb2 translation and thus leads to the downregulation of E-cadherin [45].

Another lncRNA implicated in translational regulation and EMT is the Translational Regulatory lncRNA (treRNA). TreRNA is upregulated in lymphonode metastasis in breast cancer and its expression is associated with invasive behavior *in vitro* and *in vivo* [46]. Further analysis showed that treRNA binds to EIF4G to suppress E-cadherin translation, in a 3′-UTR dependent manner [46]. This last one might be an example of the fourth category.

One further example in this (fourth?) category is the lncRNA neighbour of *BRCA1*gene 2 (*NRB2*), that is induced under energy stress [47]. *NRB2* interacts with AMPK to promote its activity. Downregulation of *NRB2* results in unchecked cell cycling/apoptotic responses and increased tumour development in xenograft models [47].

## CONCLUSIONS

Since stress response pathways are essential to restore cellular homeostasis, their alteration and misuse are at the origin of pathologies like cancer development/progression [3] and neurodegenerative disorders [48]. The importance of ribosome biogenesis and translational regulation in a pathological context, is corroborated by the number of compounds targeting these processes currently in the clinic [49].

Given its essential roles in normal cells, however, modulating translation and ribosome biogenesis in a targeted manner remains a major challenge. Importantly, lncRNAs often exhibit cell-specific expression patterns [50, 51] and therefore they would be attractive “druggable” targets.

While the dark side of the genome comes to light, new examples of lncRNAs regulating ribosome biogenesis and protein synthesis emerge. It is very likely that the mechanisms described in this review are only the tip of the iceberg and new mechanisms will be soon uncovered and described. LncRNAs could for instance affect the recognition of uORFs, affect the modification pattern of rRNAs and mRNAs, help the recruitment of specific sets of RNAs to the ribosome, thus contributing not only to global regulation of ribosomal functions, but also to the heterogeneity of the ribosome population. A systematic study of lncRNAs implicated in ribosome biogenesis and translational reprogramming would ultimately help clarifying the reasons for the association of these molecules with the ribosomes and their inability to code, thus opening new possibilities in translational research.

## Abbreviations:

DFC: dense fibrillar components,
FC: fibrillar center,
GC: granular component,
IGS: intergenic spacer,
IRES: internal ribosome entry site,
lncRNA: long non-coding RNA,
mTOR: mammalian target of rapamycin,
NoDS: nucleolar detention sequence,
rRNA: ribosomal RNA,
TORC: TOR complex,
treRNA: translational regulatory lncRNA,
uORF: upstream open reading frame,
UTR: untranslated region.
